# Inhibition of transforming growth factor-beta by Tranilast reduces tumor growth and ameliorates fibrosis in colorectal cancer

**DOI:** 10.17179/excli2020-2932

**Published:** 2021-03-09

**Authors:** Milad Hashemzehi, Negar Yavari, Farzad Rahmani, Fereshteh Asgharzadeh, Atena Soleimani, Neda Shakour, Amir Avan, Farzin Hadizadeh, Maryam Fakhraie, Reyhaneh Moradi Marjaneh, Gordon A. Ferns, Parham Reisi, Mikhail Ryzhikov, Majid Khazaei, Seyed Mahdi Hassanian

**Affiliations:** 1 Department of Medical Physiology, School of Medicine, Mashhad University of Medical Sciences, Mashhad, Iran; 2 Tropical and Communicable Diseases Research Centre, Iranshahr University of Medical Sciences, Iranshahr, Iran; 3 Department of Medical Physiology, School of Medicine, Isfahan University of Medical Sciences, Isfahan, Iran; 4 Department of Clinical Biochemistry, School of Medicine, Mashhad University of Medical Sciences, Mashhad, Iran; 5 Department of Medicinal Chemistry, School of Pharmacy, Mashhad University of Medical Sciences, Mashhad, Iran; 6 Student Research Committee, Mashhad University of Medical Sciences, Mashhad, Iran; 7 Metabolic Syndrome Research Center, Mashhad University of Medical Sciences, Mashhad, Iran; 8 Student Research Committee, School of Medicine, Mashhad University of Medical Sciences, Mashhad, Iran; 9 Biotechnology Research Center, Pharmaceutical Technology Institute, Mashhad University of Medical Sciences, Mashhad, Iran; 10 Brighton & Sussex Medical School, Division of Medical Education, Falmer, Brighton, Sussex BN1 9PH, UK; 11 Saint Louis University, School of Medicine, St. Louis, MO, USA

**Keywords:** colon cancer, Tranilast, TGF pathway, angiogenesis, fibrosis

## Abstract

Transforming Growth Factor-beta (TGF-β) is dysregulated in colorectal cancer and there is growing evidence that it is associated with a poor prognosis and chemo-resistance in several malignances, including CRC. In this study we have explored the therapeutic potential of targeting TGF-β using Tranilast in colon cancer. The anti-proliferative activity of Tranilast was evaluated in 2- and 3-dimensional cells. We used a xenograft model of colon cancer to investigate the activity of Tranilast alone or in combination with 5-FU on tumor growth using histological staining and biochemical studies, as well as gene expression analyses using RT-PCR and Western blotting. Tranilast alone or in combination with 5-FU inhibited tumor growth and was associated with a reduction of TGF-β expression and CD31 positive endothelial cells. Histological evaluation showed that Tranilast increased tumor necrosis and reduced tumor density and angiogenesis. Tranilast increased MDA and ROS production. It was also found that Tranilast reduced total thiol concentration and reduced SOD and catalase activity. Tranilast plus 5-FU was also found to attenuate collagen deposition, reducing tumor fibrosis in tumor xenografts. Our results show that Tranilast, a TGF inhibitor, in combination with 5-FU reduces tumor growth by inhibiting fibrosis and inducting ROS, thus supporting this therapeutic approach in CRC treatment.

## Introduction

Colorectal cancer (CRC) is one of the most prevalent cancers, being the fourth most common cause of cancer-related death globally (Marjaneh et al., 2018[19]). In the United States, 135,430 new colon cancer patients were identified of whom 50,260 died in 2017. There are several risk factors for CRC that include: smoking, obesity, inheritance, and inflammatory bowel disease. The incidence of CRC increases with age, and approximately 58 % of new cases are found in people over 65 years of age (Siegel et al., 2017[32]). The challenge in the treatment of colon cancer patients is finding new drugs with high efficacy and low side effects. Efforts are being made to identify and target the main pathways that contributed to the progression of cancer. 

The TGF-β pathway is a multifunctional pathway which has been reported to be up-regulated in advanced-stages of some tumors, and plays an important role in regulating the proliferation, differentiation, survival, and migration of tumor cells and angiogenesis (Gold, 1999[10]). The initiating step is the ligation of TGF-β with the type II receptor (TβRII), followed by recruitment of the type I receptor (TβRI) that is phosphorylated, and the activation of SMAD proteins, that regulate several downstream genes (Siegel and Massagué, 2003[31]). The pro-oncogenic effects of TGF-β also depend on the activation of other, non-SMAD pathways (Derynck and Zhang, 2003[6]; Zhang, 2009[41]). Therefore, TGF-β may affect tumor development through various mechanisms. It therefore has been proposed that targeting TGF-β signaling may result in significant improvements in cancer patients.

Tranilast is an inhibitor of TGF-β secretion, and has been used in the treatment of asthmatic patients (Rogosnitzky et al., 2012[25]). It has been shown that Tranilast can inhibit tumor growth and tumor cell proliferation in several different cancers including breast, pancreas, prostate and glioma (Yashiro et al., 1997[38]; Hiroi et al., 2002[13]; Shime et al., 2002[27]). There are several studies that have investigated the molecular mechanisms that contributed to the anti-proliferative effects of Tranilast. It is a TGF-β blocker, that can inhibit the production of chemokines by mast cells, and suppressed the secretion of the matrix metalloproteases (Platten et al., 2001[23]; Shimizu et al., 2005[28]; Izumi et al., 2009[16]). Tranilast has also been reported to reduce angiogenesis by inhibiting endothelial cell proliferation, reducing vascular endothelial growth factor (VEGF) expression, and endothelial tube formation (Isaji et al., 1997[15]). In this current *in vitro* and experimental animal model study we evaluated the anti-cancer potential of Tranilast alone and in combination with Fluorouracil (*5*-*FU), *revealing its underlying molecular mechanisms of action in colon cancer.

## Material and Methods

### Cell culture

CT-26 cells, a mouse colon cancer cell line, were grown in DMEM-F12 media supplemented with 10 % (v/v) FBS and penicillin/streptomycin antibiotics. Cultures were maintained at 37 °C in a humidified incubator containing 5 % CO_2_.

### Growth inhibition (MTT) assay

Cell viability was assessed using a 3-(4,5-demerthylthiazol-2-yl)-2,5-diphenyltetrazolium bromide (MTT) assay as described by Zhang et al. (2015[40]). CT-26 cells were treated with different concentration of Tranilast and the IC_50_ was determined. Each cell viability assay was performed in triplicate.

### 3D model

Spheroid formation (3D culture) was induced as described by Korff and Augustin (1998[18]) and Walzl et al. (2014[36]). CT-26 cells were seeded onto 96 well-plates in DMEM-F12 medium and treated with Tranilast (200 µM). Spheroids were grown under standard conditions and harvested at different time points for drug testing. Spheroid morphology was evaluated microscopically. 

### Western blotting

Western blotting was performed as described previously (Hassanian et al., 2015[12]). Following treatment with Tranilast (200 µM), Cyclin D1 protein was extracted using lysis buffer. We evaluated protein concentration using the Pierce bicinchoninic acid (BCA) protein assay kit (Thermo Scientific, Rockford, IL). Following sample loading and separation by SDS-PAGE, proteins were transferred onto poly vinylidene difluoride (PVDF) membranes (Immobilon-P, Millipore, Bedford, MA). We incubated PVDF membranes with primary and secondary antibodies, and visualized data using a West *Femto Chemiluminescent *reagent (Thermo Scientific, Rockford, IL).

### Reactive oxygen species (ROS) detection

We measured the production of reactive oxygen species (ROS) in the CT-26 cell line using a ROS detection assay kit (Abcam, Cambridge, MA) based on manufacturer's instructions. CT-26 cells were seeded in a 96-well culture plate. After adding DCFDA solution, cells were treated with two doses of Tranilast (100 and 200 µM) and compared with the control group. The fluorescent intensity in all groups was quantified using a fluorescence plate reader FACScan (Becton Dickinson, San Jose, CA).

### Animal study

To investigate Tranilast's effect on tumor growth *in vivo*, twenty BALB/C mice were purchased at the age of 6-8 weeks from the Pasteur Institute. Following tumor implantation, when the size of the tumor reached 80-100 mm^3^, mice were categorized into the following groups: 1) control group; 2) treated with 5-FU (5 mg/kg/day); 3) treated with Tranilast (200 µM directly intra tumor); 4) treated with 5-FU and Tranilast combination. Tissue samples were collected on day 21 and stored in formalin or at −70 °C for histological and biochemical experiments.

### Histological assay

Fixed tissue samples were embedded in paraffin and then sectioned in 5-7 μm thickness. The tissue sections were de-paraffinized and stained with Hematoxylin-Eosin (H&E) for the evaluation of tumor density, necrosis and tumor vessels. Masson trichrome staining was performed for the evaluation of collagen deposition and fibrosis.

### Measurement of oxidative stress markers

As we previously described (Asgharzadeh et al., 2017[1]; Hashemzehi et al., 2018[11]; Marjaneh et al., 2018[19]), the activity of catalase and superoxide dismutase (SOD), as well as the concentration of malondialdehyde (MDA), total thiol and nitric oxide were measured in tissue homogenates.

### Docking study

The orientation of the Tranilast at the active site of the target proteins was examined using the Molecular Operating Environment (MOE, Chemical Computing Group Inc. Montreal, [link:http://www.chemcomp.com*http://www.chemcomp.com]) docking software *in silico*. In this study we used the chemical structure drawing program, ChemDraw Ultra 7.0, and molecular modeling counterpart, Chem3D Pro 7.0, to draw and minimize the energy of Tranilast. We downloaded protein crystal structures from the RCSB Protein Data Bank. The PDB ID used in this study were 3hng (VEGFR1), 3v2a (VEGFR2), 4bsk (VEGFR3), 3hmm (ALK5), 1khx (SMAD2), 1mk2(SMAD3), 1ygs (SMAD4), 3v2a (VEGFA), 2vwe (VEGFB), 4bsk (VEGFC), 2xv7 (VEGFD), 3v6b (VEGFE), 5ty4 (TBRII), 5cvb (Col1A1), 5cti (Col1A2), and (5mjy) SARA. All the computational procedures were carried out using MOE. The docking procedure was performed with the default settings of the MOE-DOCK. The final docking scores were evaluated using the GBVI/WSA dG scoring function with the Generalized Born solvation model (GBVI) (Wojciechowski and Lesyng; 2004[37]). The GBVI/WSA dG is a force field-based scoring function, which estimates the free energy of binding of the ligand from a given pose. The dissociation constant (Ki) was computed through the binding free energy estimated with the GBVI/WSA dG scoring function according to the following equation: **Δ*G*=*RTLn*(*K_i_*)** where R represented the gas constant and T the temperature in kelvin. The Ki was computed from the binding free energy values at a fixed temperature (298 K).

### Real-Time Polymerase-Chain-Reaction (RT-PCR)

As we have previously described (Rahmani et al., 2019[24]), total RNA was isolated from Tranilast-treated tissues. First-strand cDNA synthesis and amplification were done using QuantiTect Reverse Transcription Kit (Qiagen, Germany). Real time-PCR reactions were performed using primers specific for TGF-β, Cyclin D1, VEGF, VEGF receptor. All experiments were performed in triplicate.

### Immunohistochemistry (IHC) assay

Immunohistochemistry staining of platelet-endothelial cells adhesion molecule (CD31) was performed to evaluate tumor angiogenesis in tissue samples. In summary, de-paraffinized sections were re-hydrated in alcohols. Slides were heated in EDTA + PBS for 20 minutes. Hydrogen peroxide (H_2_O_2)_ was used for the inhibition of endogenous per- oxidase. Incubation with primary and secondary antibodies were performed. Then sections were treated with DAB solution and hematoxylin was used for staining. Finally, sections were dehydrated and vessel density was evaluated using light microscope.

### Statistical analysis

Statistical analysis was conducted using one-way analysis of the variance (ANOVA) and t-test. All statistical analyses were done using SPSS software (version 16.0) and the data were expressed as mean ± SEM. In all cases, p < 0.05 was considered significant.

## Results

### Tranilast inhibits cell growth

**Figure 1:**
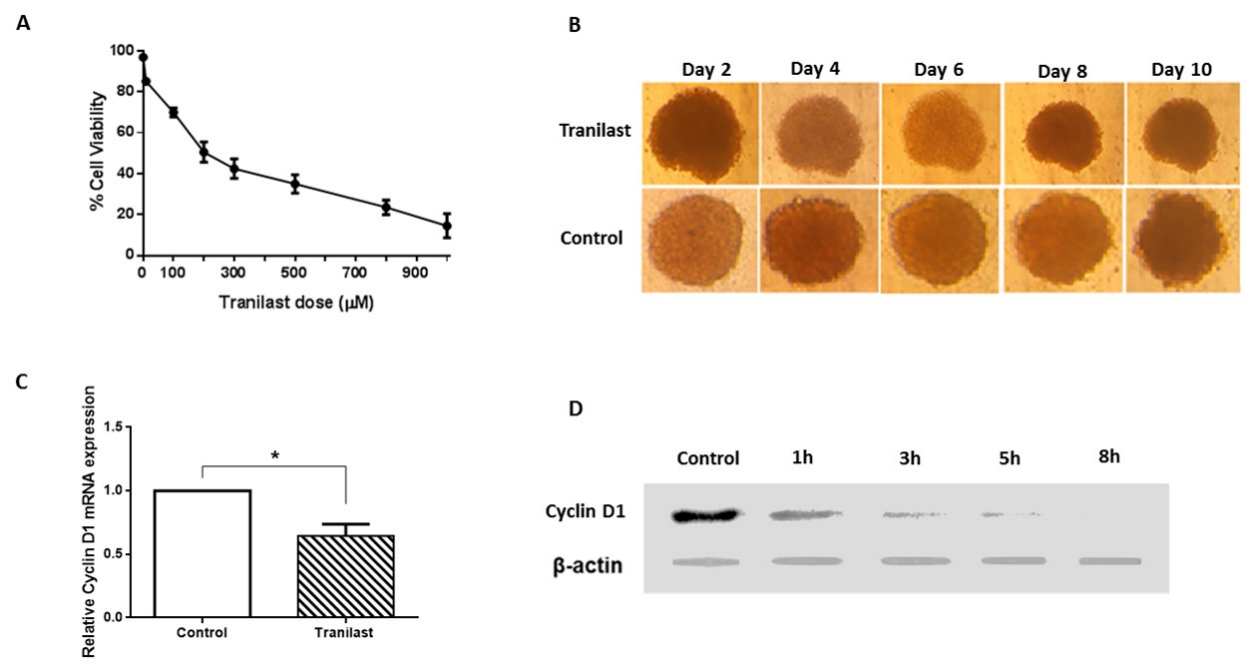
Tranilast inhibited colon cancer cell viability. (A) Tranilast dose-dependently reduced the CT-26 cell viability. (B) Spheroid formation was reduced in response to Tranilast treatment. (C-D) The effect of Tranilast in Cyclin D1 expression in mRNA and protein levels *in vivo* and *in vitro*, respectively. *p<0.05

To determine Tranilast toxicity, the MTT analysis was performed in CT-26 colon cancer cell lines. Tranilast was found to reduce CT-26 cell viability in a dose-dependent manner (Figure 1A(Fig. 1)) with an IC_50_ value of 200 µM. Moreover, the studies in the cell spheroids showed that Tranilast time-dependently suppressed the formation of sphere colonies (Figure 1B(Fig. 1)).

There are several studies reporting a positive correlation between Cyclin D1 over-expression and malignancies including colon cancer. Amplification of this gene has an important role in cell cycle progression, which frequently occurs in various tumors contributing to tumorigenesis (Casimiro et al., 2014[3]; Zhao et al., 2014[42]). Our results showed that Tranilast significantly decreased mRNA and protein expression of Cyclin D1 *in vivo* (Figure 1C(Fig. 1)) and *in vitro *(Figure 1D(Fig. 1)).

### Tranilast reduced tumor growth in xenograft model

**Figure 2:**
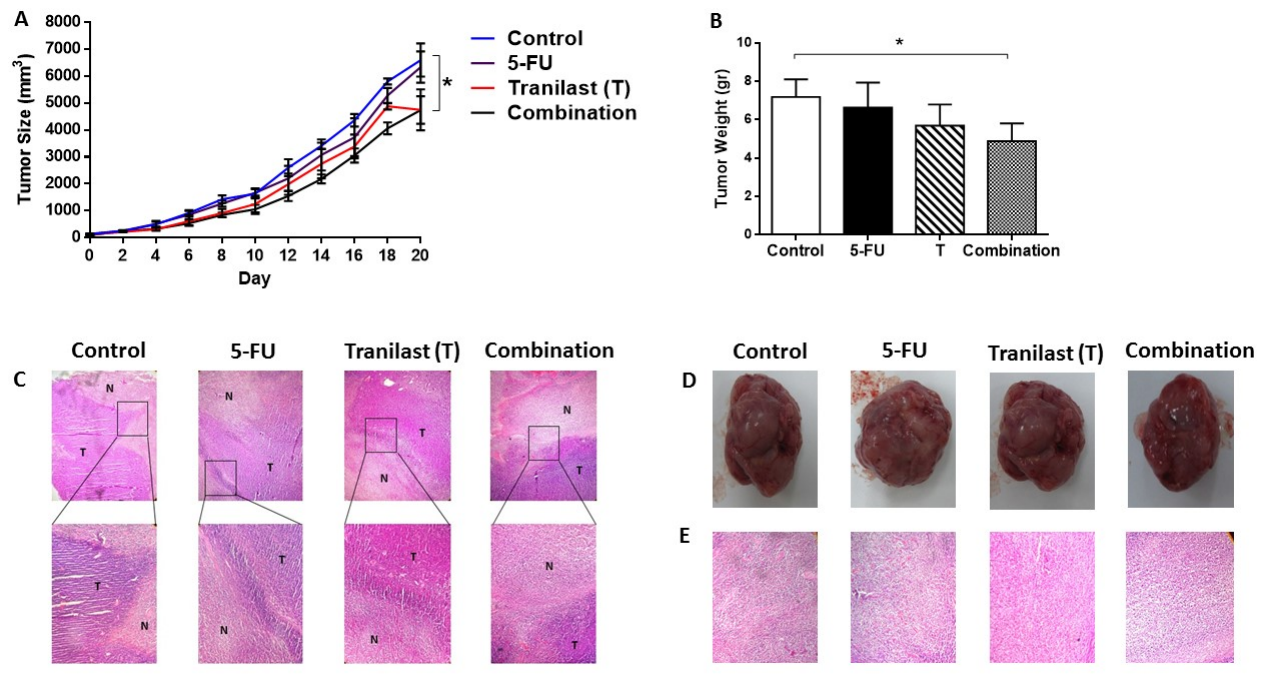
Tranilast reduced tumor growth in xenograft model. (A-B) Tumor size (A) and tumor weight (B) were reduced in treated groups. (C-E) H&E staining of tissue sample (х10) and (х40). Tissue staining showed elevation of tumor necrosis (C) and reduction of tumor density (D-E) in treated groups. Tumor tissue showed tumor cells (T), necrotic area (N). *p<0.05

To further evaluate the anti-cancer potential of Tranilast, we developed a xenograft model of colon cancer. Results show that Tranilast (200 µM) alone or in combination with 5-FU is an effective chemotherapy compound, significantly inhibiting tumor growth compared to control and 5-FU groups. The time course of tumor size is shown in Figure 2A(Fig. 2). However, the inhibitory effect of Tranilast on tumor weight was observed, but these effects were only found to be significant when used in combination with 5-FU (Figure 2B(Fig. 2)). Consistent with these results, histological evaluation showed a higher frequency of tumor necrosis (Figure 2C(Fig. 2)) and lower tumor density (Figure 2D-E(Fig. 2)) in treatment groups. These findings represent Tranilast's efficacy in reducing carcinogenic properties in the colon cancer mice model.

### Analysis on the interaction of Tranilast with TGF-β, angiogenesis and fibrosis

**Figure 3:**
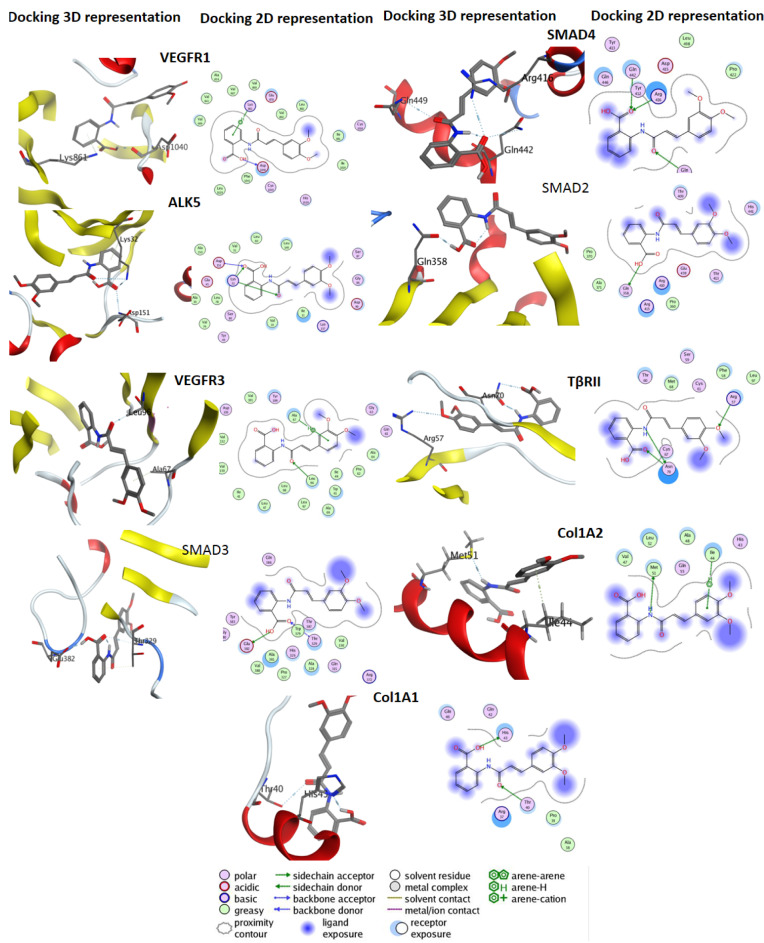
Bioinformatic analysis on the interaction of Tranilast with TGF-β, angiogenesis and fibrosis. The orientation of the Tranilast in active site of the target proteins was examined by Molecular Operating Environment (MOE, Chemical Computing Group Inc. Montreal, http://www.chemcomp.com docking experiment. The chemical structure drawing program, ChemDraw Ultra 7.0, and molecular modeling counterpart, Chem3D Pro 7.0 was used to draw and minimize the energy of Tranilast. The final docking scores were assessed by the GBVI/WSA dG scoring function with the Generalized Born solvation model.

We studied the affinity of Tranilast to proteins involved with TGF-β, angiogenesis and fibrosis. Docking results indicated that this drug directly interacts with all of the mentioned proteins (Figure 3(Fig. 3), Table 1(Tab. 1), Supplementary Figure 1). Among these proteins, VEGFR1 had a pKi = 5.11 through one H donor bonding an oxygen of a Tranilast̓'s hydroxyl group to oxygen of ASP_1040 residue of Vascular Endothelial Growth Factor of receptor 1 with a distance of 3.00 Å and pi-H binding of one of its benzene groups to carbon of LYS_861 with a distance of 3.87 Å, followed by ALK5 (pKi = 4.62; three H acceptor bonding of the ligand's carbonyl group oxygen to nitrogens of LYS_32, ASP_151, LYS_32 residues with distances of 2.79 Å, 3.04 Å and 3.84 Å; VEGFR3 (pKi = 4.24; one H acceptor bonding of ligand's carbonyl group oxygen to carbon of LEU_96 residue with a distance of 3.18 Å, and one pi-H binding of one benzene group to a carbon of ALA_67 residue with a distance of 3.54 Å; SMAD3 (pKi = 3.78; two H donor bonding of ligand's hydroxyl group hydrogen to oxygen of GLU_382 residue with a distance of 2.79 Å, and of its carbonyl group oxygen to carbon of THR_329 residue with a distance of 3.36 Å; SMAD4 (pKi = 3.64; three H acceptor binding of ligand's carbonyl group oxygen to nitrogen atom nitrogen of ARG_416, GLN_442, and GLN 449(N) residues with distances of 3.34 Å, 3.75 Å, and 3.54 Å, respectively; VEGF-A (pKi = 3.60; one H donor binding of its hydrogen atom attached to nitrogen to oxygen of CYS_61 residue with a distance of 3.57 Å; SMAD2 (pKi = 3.57; one H donor binding of ligand's hydrogen attached oxygen of hydroxyl group to oxygen of GLN_358 residue with a distance of 3.12 Å; VEGF-D (pKi = 3.45; one H acceptor binding between carbonyl group oxygen and oxygen of THR_130 residue with a distance of 3.26 Å; VEGF-C (pKi = 3.40, two H donor bonding among hydrogen of hydroxyl group to sulfur of CYS_211, and its hydrogen-attached nitrogen to carbonyl group oxygen of ASN_167 residue with distances of 3.70 Å and 3.23 Å, and one H acceptor binding one of ligand's carbonyl group oxygen to carbon of one SER_168 residue with a distance of 3.68 Å; VEGF-B (pKi = 3.31; one acceptor of a carbonyl group oxygen with nitrogen atom of LYS_45 residue with a distance of 3.39 Å; VEGFR2 (pKi = 3.28; one H acceptor binding of its carbonyl group oxygen to nitrogen atom of TRP_179 residue with a distance of 3.14 Å and one pi-H binding of its benzene ring to carbon of ASN_158 residue with a distance of 3.74 Å; TβRII (pKi = 3.16; one H donor binding one of ligand's nitrogen atoms to carbonyl group oxygen of ASN_70 residue with a distance of 2.63 Å and two H acceptors binding an oxygen of a carbonyl group to a hydrogen attached nitrogen to ASN_70 residue with a distance of 2.96 Å, and among one of its hydroxyl oxygens to a nitrogen atom of ARG_57 residue with a distance of 3.32 Å; Col1A2 (pKi = 3.15; one H donor binding of a hydrogen-attached nitrogen atom of ligand to sulfur of MET_51 residue with a distance of 3.88 Å, and one pi-H binding among one of its benzenes to carbon of ILE_44 residue with a distance of 3.72 Å; VEGF-E (pki = 3.14; two H donor binding of a ligand's hydroxyl group oxygen to carbonyl group oxygen of ASN_62 residue, and a hydrogen attached nitrogen of Tranilast to sulfur atom of CYS_60 residue with a distances 3.18 Å, and 4.21 Å; Col1A1 (pKi = 2.82; one H donor binding of a hydrogen of ligand's hydroxyl group to nitrogen atom of HIS_43 residue with a distance 2.97 Å, and one H acceptor binding a carbonyl group oxygen to oxygen of THR_40 residue with a distance of 3.28 Å, and SARA (pKi = 2.73, one H acceptor binding among Tranilast's carbonyl group oxygen to nitrogen atom of LYS_14 43 residue with a distance 3.06 Å (Table 1(Tab. 1)). According to the above-mentioned results, receptors such as VEGFR1, ALK5, VEGFR3 with pKi ≥ 3.57 were found to have strong binding to Tranilast, indicating that Tranilast has substantial affinity for these receptors. Among them, VEGFR1, with a pKi value of 5.11, was shown to have stronger binding to Tranilast compared to other proteins (Figure 3(Fig. 3)). 

### Tranilast decreased angiogenesis and fibrosis

**Figure 4:**
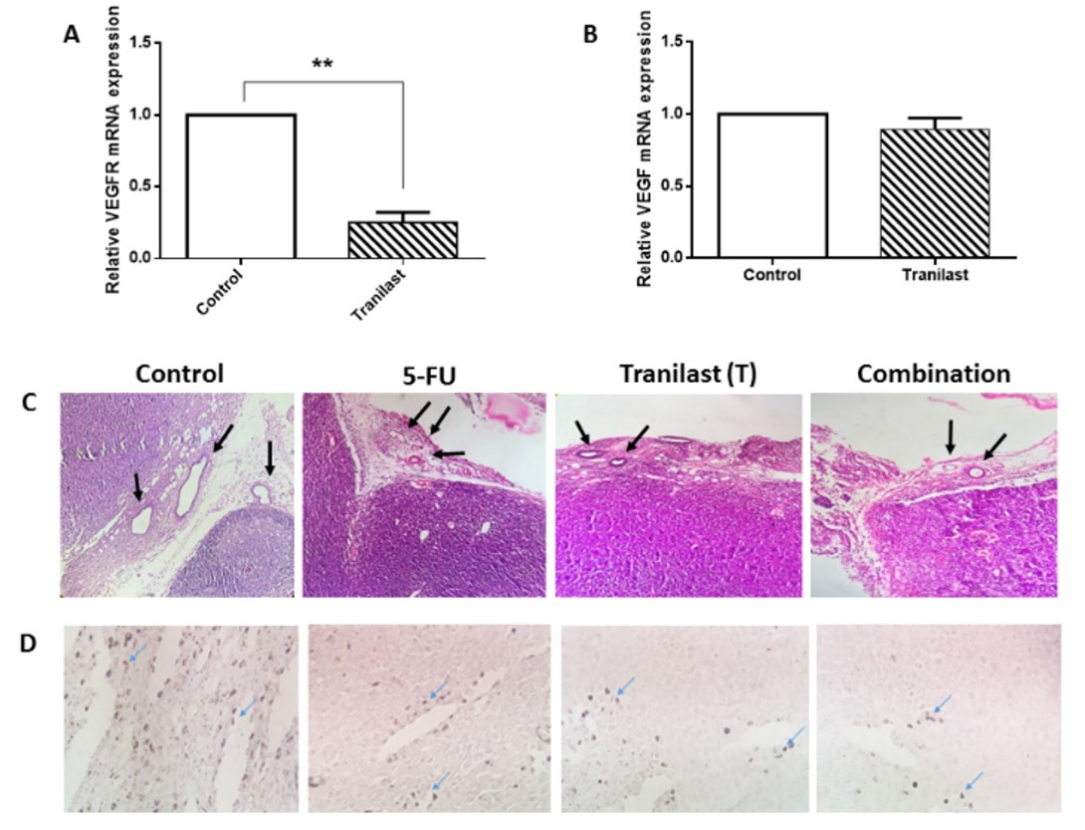
Tranilast decreased angiogenesis in tumor tissue. (A) H&E staining of vascular disruption. Vascular formation was decreased in treated groups (Arrows). (B) Immunohistochemistry staining of CD-31 positive endothelial cell

**Figure 5:**
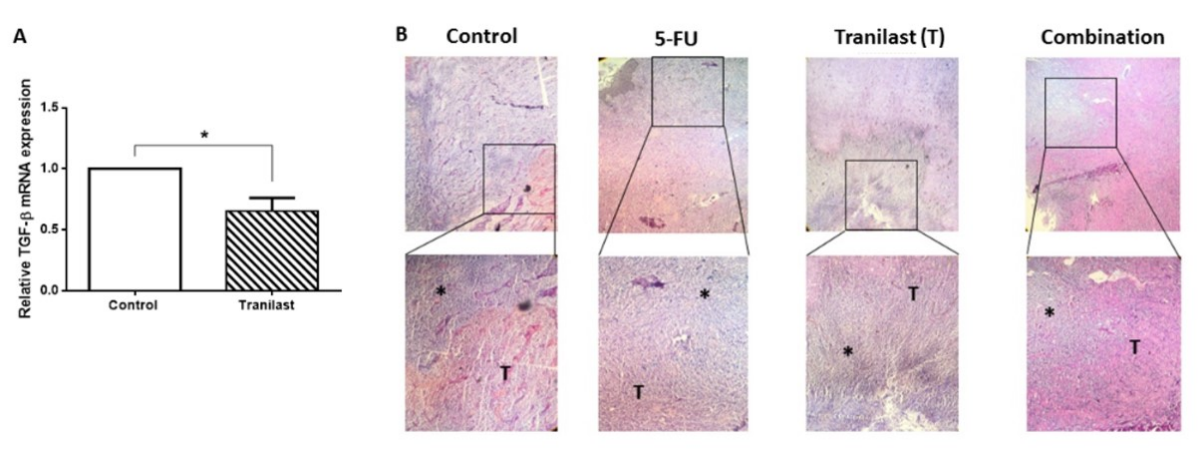
Tranilast reduced fibrosis in colon cancer. (A) Tranilast reduced the mRNA expression of TGF-β in tissue samples. (B) Trichrome staining of tissue sample. *p<0.05; ***p<0.001

Tumor vascularization is a general requirement for tumor development and invasiveness (Döme et al., 2007[7]). Studies have previously shown that targeting VEGF or VEGFR could prevent angiogenesis, indicating that targeting VEGF and its receptor may benefit therapeutic strategies (Eskens and Verweij, 2006[8]). Consistently, we showed that Tranilast inhibited mRNA expression of VEGFR in colon cancer tissues. VEGF showed no significant response to Tranilast (Figure 4A-B(Fig. 4)). We assessed whether Tranilast alone or in combination with 5-FU could impact vascularization in tumor tissues. Similar to previous data, Tranilast was able to alleviate tumor features via suppression of vascular formation in tissues samples (Figure 4C(Fig. 4)). As we have shown, it also decreased the CD31 positive endothelial cell count, as one of the angiogenic factors, reducing the capillary density in specimens (Figure 4D(Fig. 4)). Several studies have reported that TGF-β, is an oncogenic mediator contributing to colon cancer progression (Soleimani et al., 2019[33]). Here we have shown that Tranilast treatment could reduce TGF-β mRNA expression in colon cancer tissues (Figure 5A(Fig. 5)). In addition, histological evaluation confirmed that combination administration attenuated collagen deposition and reducing tumor fibrosis in tumor xenograft tissues (Figure 5B(Fig. 5)). 

### Regulatory effect of Tranilast on oxidant/ antioxidant status

**Figure 6:**
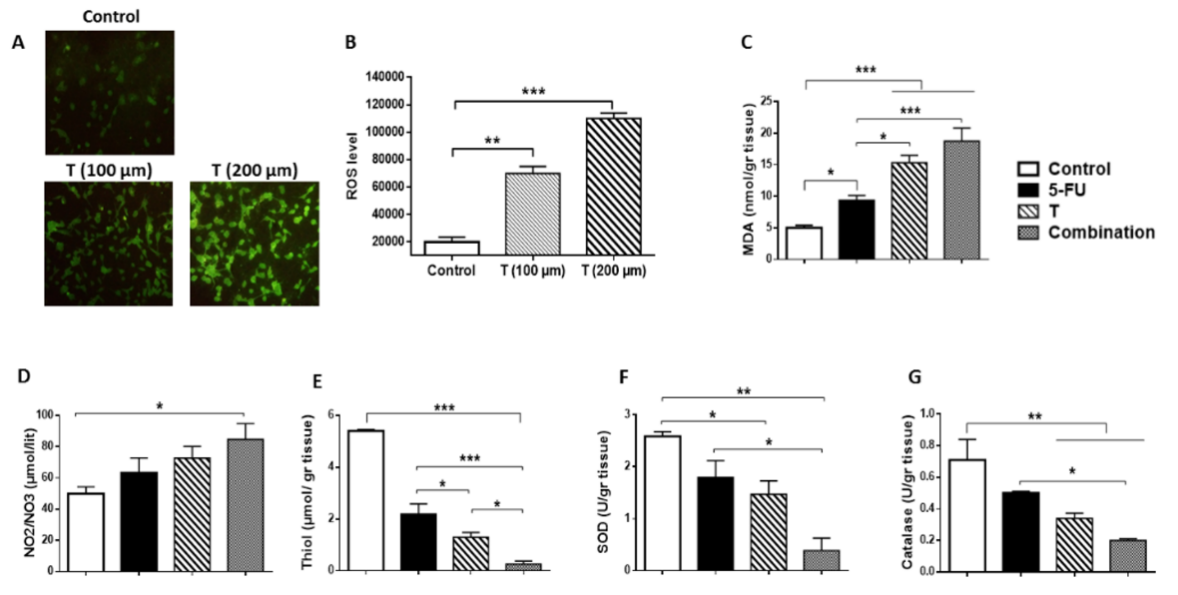
Modulating effect of tranilast in oxidant/antioxidant status. (A-B) Tranilast increased the production level of ROS in CT-26 cell lines in a dose-dependent manner. (C-G) Comparison of the concentration of MDA (C), Nitric Oxide (D), and total Thiol (E) levels and SOD (F), and Catalase (G) enzyme activity in tissue homogenates. *p<0.05; **p<0.01; ***p<0.001

Disruption in the oxidant/antioxidant equilibrium results in oxidative stress which has a critical role in colon cancer progression (Chang et al., 2008[4]). In the next step we investigated the oxidant/antioxidant effects of Tranilast *in vitro* and *in vivo*. Our results indicated that Tranilast dose-dependently significantly increased the level of ROS production in CT-26 cell line (Figure 6A-B(Fig. 6)). It has also shown that Tranilast alone or in combination with 5-FU could elevate the MDA (Figure 6C(Fig. 6)) and nitric oxide (Figure 6D(Fig. 6)) levels, while total thiol concentration (Figure 6E(Fig. 6)), superoxidase dismutase (SOD) (Figure 6F(Fig. 6)) and catalase activities (Figure 6G(Fig. 6)) were decreased in tissue homogenates.

## Discussion

In the current study, we have evaluated the potential therapeutic effect of Tranilast as a TGF-β inhibitor in colon cancer. We have shown that Tranilast is capable of suppressing cell cycle progression and regulate oxidative stress condition in CT-26 cell line. Our results indicate that Tranilast reduces the growth of tumor cells which was accompanied by a reduction in angiogenesis and fibrosis. Along with these effects, Tranilast modulated the oxidant-antioxidant status in tissue samples. 

There is growing evidence that TGF-β is involved in tumorigenesis which is discussed in our previous papers (Khoshakhlagh et al., 2019[17]; Soleimani et al., 2019[33]). In the early stage of tumor progression, TGF-β acts as a tumor suppressor while during tumor development process, cells became resistant to tumor growth inhibitory effects of TGF-β (Fink et al., 2001[9]). In interest of our work, non-physiological secretion of TGF-β in advanced stages increased tumor growth in tumor cells and environment via autocrine/paracrine manner, which is associated with activation of SMAD and non-SMAD pathways (Moustakas et al., 2002[20]). Zhang et al. have reported that inhibition of the TGF-β signaling pathway could attenuate the invasive properties and improved survival in mice colon carcinoma CT-26 cell lines (Zhang et al., 2009[39]). Therefore, using a pharmaceutical inhibitor against oncogenic effects of TGF-β may be useful therapeutically for colon cancer treatment.

Results of cell proliferation assay showed that Tranilast could reduce proliferation of pancreatic cancer cells leading to a decrease of tumor growth (Hiroi et al., 2002[13]). Similarly, Izumi et al., showed that Tranilast stimulated cell cycle arrest and apoptotic cell death *in vitro* and inhibited the tumor growth in experimental mice model in prostate cancer (Izumi et al., 2009[16]). The clonogenicity findings also represented a significant reduction in the number and size of colonies following Tranilast treatment in breast cancer cell lines (Subramaniam et al., 2011[34]). In line with these results, our data showed that Tranilast reduced tumor size, weight and density and increased necrosis in colon cancer mice model. 

Interaction between cancer associated fibroblast (CAF) and cancer cells which mediated through multiple factors including TGF-β is a key determinant in tumor progression (Semba et al., 2009[26]; Shinto et al., 2010[30]). It has been shown that inhibition of the TGF-β pathway attenuates the epithelial-mesenchymal transition (EMT) and fibrosis in cancer (Shinto et al., 2010[30]; Tsukada et al., 2013[35]; Shinbo et al., 2015[29]). Previous studies have demonstrated that Tranilast decreased CAF function, thereby inhibiting fibrosis (Darakhshan and Pour, 2015[5]). Consistently, we indicated that Tranilast decreased TGF expression in CT-26 cell line. Moreover, a significant reduction of collagen deposition and fibrosis area was observed in treated groups *in vivo*. 

Angiogenesis is a multi-step process that is involved in tumor progression. Both vascular endothelial growth factor (VEGF) and tumor cell-stimulated transforming growth factor (TGF) contribute to angiogenesis (Ikeda et al., 2001[14]). It has been shown that Tranilast significantly reduced microvessel density in metastatic pancreatic cancer (Hiroi et al., 2002[13]). Furthermore, Isaji et al., demonstrated that Tranilast reduced proliferation and VEGF-induced chemotaxis and tube formation of endothelial cells (Isaji et al., 1997[15]). In agreement with these results, we demonstrated that Tranilast is able to reduce vascular density, and CD-31 expression which is a marker of endothelial cell.

There is growing evidence that ROS production is one of the mechanisms involved in current therapeutic strategies which are associated with a reduction of cell proliferation and viability (Negrei et al., 2016[21]). Bernardes et al., have demonstrated a negative relationship between the serum levels of TGF-β1 and the levels of malondialdehyde (MDA) and advanced oxidation protein products (AOPP) in melanoma. They also showed that TGF-β1 increased antioxidant Glutathione (GSH) level in melanoma patients (Bernardes et al., 2016[2]). Similar to these results, Panis et al., indicated a positive relation between TGF-β1 and antioxidant marker level in breast cancer (Panis et al., 2013[22]). To further investigate the mechanism of Tranilast performance, we also compared the oxidant/anti-oxidant balance in different treatment groups in tissue samples and CT-26 cell line. Consistent with previous papers, we demonstrated that inhibition of TGF-β production via Tranilast modulated oxidant/antioxidant status in colon cancer. 

Taken together, our results indicated that Tranilast, a TGF pathway inhibitor, alone or in combination with 5-FU could reduce tumor growth and invasiveness through the inhibition of angiogenesis and fibrosis and modulating oxidative stress condition. However, the combination treatment was more potent. Comprehensive preclinical and clinical studies are needed to further explore the therapeutic potential of Tranilast and its mechanisms of action in different conditions.

## Notes

Milad Hashemzehi, Negar Yavari and Amir Avan contributed equally as first authors.

Majid Khazaei and Seyed Mahdi Hassanian (Department of Clinical Biochemistry, School of Medicine, Mashhad University of Medical Sciences, Mashhad, Iran; Phone: (+98) 5138002375, Fax: (+98) 5138002389, E-mail: hasanianmehrm@mums.ac.ir) contributed equally as corresponding authors.

## Funding

This study was supported by grants awarded by the Mashhad University of Medical Sciences, the Biotechnology Development Council of the Islamic Republic of Iran (Grant No. 970306), and National Institute for Medical Research Development.

## Conflicts of interest

The authors have no conflicts of interest.

## Supplementary Material

Supplementary data

## Figures and Tables

**Table 1:**
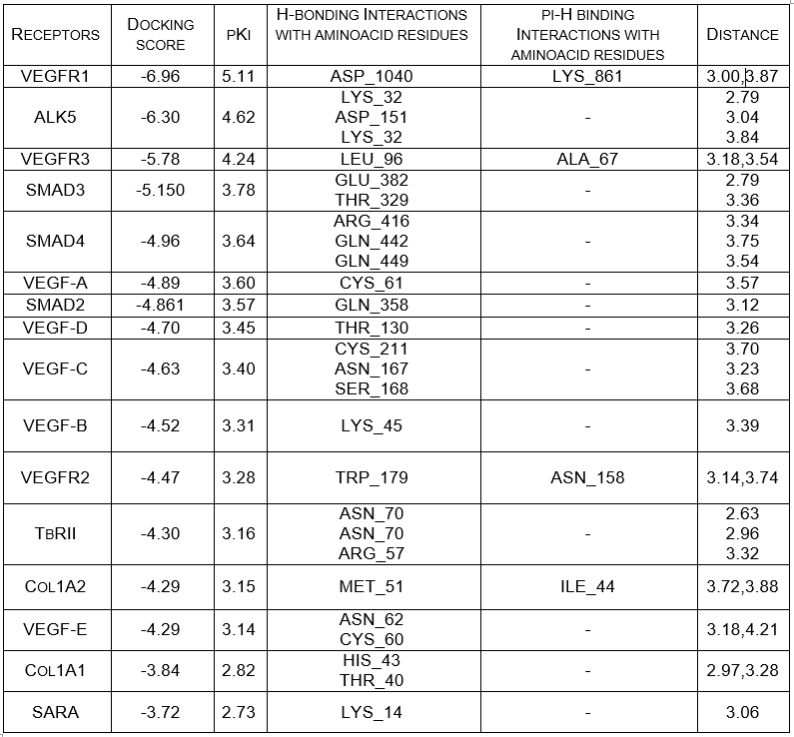
Data for the docking interactions of Tranilast at the active sites of VEGFR (VEGFR1, VEGFR2, VEGFR3), ALK5, SMAD (2,3,4), VEGF (A, B, C, D, E), TBRII, Col1A1, Col1A2, and SARA as the molecular targets
