# Supplementary material for: Inhibition of transforming growth factor-beta by Tranilast reduces tumor growth and ameliorates fibrosis in colorectal cancer
Source: EXCLI J. 2021 Mar 9;20:601–13. doi: 10.17179/excli2020-2932 (PMC8056055; doi:10.17179/excli2020-2932)
Supplement: Supplementary data [file EXCLI-20-601-s-001.pdf]

EXCLI Journal 2021;20:601-613 – ISSN 1611-2156
Received: September 21, 2020, accepted: March 03, 2021, published: March 09, 2021

Supplementary data to:

Original article:

INHIBITION OF TRANSFORMING GROWTH FACTOR-BETA BY TRANILAST REDUCES TUMOR GROWTH AND AMELIORATES FIBROSIS IN COLORECTAL CANCER

Milad Hashemzehi[1,2,*], Negar Yavari[3,*], Farzad Rahmani[2], Fereshteh Asgharzadeh[1], Atena Soleimani[4], Neda Shakour[5,6], Amir Avan[7,8,*], Farzin Hadizadeh[5,9], Maryam Fakhraie[1], Reyhaneh Moradi Marjaneh[1], Gordon A. Ferns[10], Parham Reisi[3], Mikhail Ryzhikov[11], Majid Khazaei[1,7#], Seyed Mahdi Hassanian[4,7#]

1 Department of Medical Physiology, School of Medicine, Mashhad University of Medical Sciences, Mashhad, Iran
2 Tropical and Communicable Diseases Research Centre, Iranshahr University of Medical Sciences, Iranshahr, Iran
3 Department of Medical Physiology, School of Medicine, Isfahan University of Medical Sciences, Isfahan, Iran
4 Department of Clinical Biochemistry, School of Medicine, Mashhad University of Medical Sciences, Mashhad, Iran
5 Department of Medicinal Chemistry, School of Pharmacy, Mashhad University of Medical Sciences, Mashhad, Iran
6 Student Research Committee, Mashhad University of Medical Sciences, Mashhad, Iran
7 Metabolic Syndrome Research Center, Mashhad University of Medical Sciences, Mashhad, Iran
8 Student Research Committee, School of Medicine, Mashhad University of Medical Sciences, Mashhad, Iran
9 Biotechnology Research Center, Pharmaceutical Technology Institute, Mashhad University of Medical Sciences, Mashhad, Iran
10 Brighton & Sussex Medical School, Division of Medical Education, Falmer, Brighton, Sussex BN1 9PH, UK
11 Saint Louis University, School of Medicine, St. Louis, MO, USA
* These authors contributed equally as first author.

Corresponding authors: Majid Khazaei, MD, Ph.D., Department of Medical Physiology, School of Medicine, Mashhad University of Medical Sciences, Mashhad, Iran. Phone: (+98) 5138002227, Fax: (+98) 5138002389, E-mail: KhazaeiM@mums.ac.ir
Seyed Mahdi Hassanian, Ph.D., Department of Clinical Biochemistry, School of Medicine, Mashhad University of Medical Sciences, Mashhad, Iran. Phone: (+98) 5138002375, Fax: (+98) 5138002389, E-mail: hasanianmehrm@mums.ac.ir

http://dx.doi.org/10.17179/excli2020-2932

This is an Open Access article distributed under the terms of the Creative Commons Attribution License (http://creativecommons.org/licenses/by/4.0/).

EXCLI Journal 2021;20:601-613 – ISSN 1611-2156
Received: September 21, 2020, accepted: March 03, 2021, published: March 09, 2021

Supplementary Table 1: Tranilast inhibited colon cancer cell viability (Raw data concerning Figure1A)

**Cytotoxic effects of Tranilast on CT-26 cells**			
**Tranilast doses (nM)**	CT-26 cell viability		
**0**	98.62	95.39	96.34
**10**	86.06	83.27	85.91
**100**	67.49	71.88	70.15
**200**	55.81	46.06	49.62
**300**	41.51	38.16	47.56
**500**	36.53	29.93	38.49
**800**	19.43	25.07	26.01
**1000**	8.62	14.5	20.36

Supplementary Table 2: The effect of Tranilast in Cyclin D1 expression in mRNA levels (Raw data concerning Figure1B)

**Cyclin D1 mRNA levels**			
**Control**	Tranilast		
**1**	0.56	0.8	0.73
**1**	0.71	0.62	0.51
**1**	0.6	0.57	0.73

Supplementary Table 3: The effect of Tranilast in VEGFR expression in mRNA levels (Raw data concerning Figure4A)

**VEGFR mRNA levels**			
**Control**	Tranilast		
**1**	0.18	0.4	0.29
**1**	0.33	0.27	0.16
**1**	0.2	0.38	0.31

Supplementary Table 4: The effect of Tranilast in VEGF expression in mRNA levels (Raw data concerning Figure4B)

**VEGF mRNA levels**			
**Control**	Tranilast		
**1**	0.95	0.91	0.88
**1**	0.84	0.76	0.69
**1**	0.77	0.8	0.72

Supplementary Table 5: The effect of Tranilast in TGF-β expression in mRNA levels (Raw data concerning Figure5A)

**TGF-β mRNA levels**			
**Control**	Tranilast		
**1**	0.6	0.51	0.66
**1**	0.73	0.79	0.58
**1**	0.59	0.62	0.7

EXCLI Journal 2021;20:601-613 – ISSN 1611-2156
Received: September 21, 2020, accepted: March 03, 2021, published: March 09, 2021

Supplementary Table 6: Modulating effect of Tranilast in Oxidative and nitrosative stress status (Raw data concerning Figure 6C-G)

Oxidative and nitrosative stress (Concentration)					
**Group**	MDA	Thiol	SOD	CAT	NO2/NO3
**Control**	8.012820513	4.327205882	2.697589	0.722047018	43.72011
**Control**	4.423076923	4.846470588	4.410542	0.575544725	46.08918
**Control**	5.320512821	4.201323529	2.235896	0.439506881	63.10341
**Control**	7.307692308	4.122647059	2.820892	0.786926606	46.51992
**Control**	3.782051282	5.633235294	3.495884	0.625774083	
**Control**	5.641025641	5.397205882	2.770908	0.45415711	
**Control**	5.192307692	5.318529412	2.511418	0.774369266	
**Control**	4.294871795	5.507352941	2.489069	0.925057339	
**5-FU**	8.076923077	2.423235294	1.57779	0.433228211	80.11764
**5-FU**	9.487179487	2.486176471	1.606251	0.357884174	64.39563
**5-FU**	7.820512821	2.470441176	1.853221	0.397649083	65.68785
**5-FU**	12.11538462	2.344558824	1.367525	0.397649083	95.19354
**5-FU**	6.730769231	2.533382353	1.495397	0.261611239	33.81309
**5-FU**	9.038461538	2.470441176	1.903591	0.255332569	40.70493
**5-FU**	7.371794872	2.863823529	2.338094	0.391370413	
**5-FU**	9.807692308	2.753676471	1.937928	0.416485092	
**5-FU**	7.948717949	2.596323529	2.215998	0.477178899	
**5-FU**	9.871794872	1.384705882	2.026508	0.514850917	
**5-FU**	10.38461538	2.596323529	1.16428	0.510665138	
**Tranilast +5-FU**	18.78205128	0.062941176	0.5544243	0.194638761	70.21062
**Tranilast +5-FU**	15.19230769	0.487794118	1.392194	0.188360092	104.66982
**Tranilast +5-FU**	22.37179487	0.236029412	0.8591433	0.22184633	49.10436
**Tranilast**	13.20512821	1.132941176	1.482089	0.332769495	62.02656
**Tranilast**	15.64102564	1.667941176	1.009122	0.412299312	79.90227
**Tranilast**	17.17948718	1.101470588	1.937928	0.297190367	122.54553

EXCLI Journal 2021;20:601-613 – ISSN 1611-2156
Received: September 21, 2020, accepted: March 03, 2021, published: March 09, 2021

Supplementary Table 7: Tranilast reduced tumor growth and tumor weight in xenograft model (Raw data concerning Figure 2A-B)

Tumor growth and tumor weight												
**Group**	Size 0	Size 2	Size 4	Size 6	Size 8	Size 10	Size 12	Size 14	Size 16	Size 18	Size 20	Weight
**Control**	168.78	254.63	451.25	958.81	1350.36	1944.81	2916.00	3600.00	4200.00	6000.00	7812.00	7.84
**Control**	128.56	288.00	352.00	784.00	1238.23	1600.00	1982.06	3200.00	4829.00	5800.00	6178.00	6.66
**Control**	121.00	207.83	692.22	1023.41	1708.51	1361.65	2916.00	3450.00	4061.25	5603.59	5808.00	6.56
**Control**	127.01	230.00	856.00	906.00	1562.00	1645.00	2453.00	3452.00	4223.00	5999.00	6630.00	7.02
**Control**	135.17	220.00	665.00	887.00	1789.00	1989.00	2631.00	3299.00	4531.00	5776.00	6230.00	7.20
**5-FU**	100.00	216.00	772.00	802.00	1852.00	2001.00	2133.00	3368.00	4129.00	5669.00	6541.00	6.89
**5-FU**	125.44	245.00	388.08	631.75	1171.88	1656.37	2524.06	2900.00	3179.00	5000.00	5733.00	5.76
**5-FU**	176.40	265.60	726.00	1191.64	1504.48	1968.75	2800.00	4150.00	5132.00	6665.00	7500.00	7.53
**5-FU**	118.80	280.00	437.40	742.56	1145.16	1379.00	1294.34	2176.00	3232.00	5566.00	5777.00	5.98
**5-FU**	90.75	263.00	705.00	899.00	1236.00	1895.00	1998.00	3231.00	4565.00	5962.00	6211.00	6.02
**5-FU**	78.75	255.00	699.00	908.00	1365.00	1785.00	2223.00	3114.00	4635.00	5698.00	5998.00	6.21
**Tranilast**	117.00	245.00	566.00	798.00	1298.00	1901.00	2312.00	2656.00	4212.00	5997.00	6556.00	6.33
**Tranilast**	127.01	205.80	320.00	864.00	1054.69	1800.00	2722.50	3289.54	3828.90	6050.00	4989.00	6.56
**Tranilast**	135.17	245.48	410.83	683.65	1081.67	1183.00	1861.94	2200.00	2950.00	3711.12	3992.63	4.98
**Tranilast**	100.00	196.02	211.25	309.08	614.08	724.72	1383.84	2765.00	3221.00	4235.00	4563.00	5.64
**Tranilast**	168.78	220.00	411.00	699.00	1352.00	1523.00	1556.00	2645.00	3412.00	4365.00	4653.00	5.89
**Tranilast**	128.56	223.00	387.00	499.00	1365.00	1694.00	1756.00	2563.00	3564.00	4865.00	5001.00	5.50
**Combination**	121.00	231.00	356.00	563.00	1299.00	1365.00	2315.00	2365.00	3664.00	4658.00	5003.00	5.80
**Combination**	90.75	231.04	245.48	384.00	1028.50	1322.78	1774.50	2299.92	2587.50	3900.00	4873.50	5.27
**Combination**	78.75	186.52	348.48	699.84	900.00	1098.50	1687.50	2400.00	3078.00	3790.50	3790.00	3.82
**Combination**	117.00	317.52	405.00	520.00	620.00	742.56	1179.14	1850.00	3500.00	4500.00	5566.00	5.57
**Combination**	125.44	215.00	335.00	456.00	899.00	1123.00	1236.00	1994.00	3321.00	4001.00	4556.00	4.20
**Combination**	176.40	241.00	321.00	405.00	1149.00	1231.00	1452.00	2123.00	3225.00	4102.00	4720.00	4.50
